# Inflammatory cell death, PANoptosis, screen identifies host factors in coronavirus innate immune response as therapeutic targets

**DOI:** 10.1038/s42003-023-05414-9

**Published:** 2023-10-20

**Authors:** R. K. Subbarao Malireddi, Ratnakar R. Bynigeri, Raghvendra Mall, Jon P. Connelly, Shondra M. Pruett-Miller, Thirumala-Devi Kanneganti

**Affiliations:** 1 Department of Immunology, St. Jude Children’s Research Hospital, Memphis, TN 38105 USA; 2 Center for Advanced Genome Engineering (CAGE), St. Jude Children’s Research Hospital, Memphis, TN 38105 USA; 3 Present Address: Biotechnology Research Center, Technology Innovation Institute, Abu Dhabi, P.O. Box 9639, United Arab Emirates

**Keywords:** Cell biology, Microbiology

## Abstract

The COVID-19 pandemic, caused by the β-coronavirus (β-CoV) severe acute respiratory syndrome coronavirus 2 (SARS-CoV-2), continues to cause significant global morbidity and mortality. While vaccines have reduced the overall number of severe infections, there remains an incomplete understanding of viral entry and innate immune activation, which can drive pathology. Innate immune responses characterized by positive feedback between cell death and cytokine release can amplify the inflammatory cytokine storm during β-CoV–mediated infection to drive pathology. Therefore, there remains an unmet need to understand innate immune processes in response to β-CoV infections to identify therapeutic strategies. To address this gap, here we used an MHV model and developed a whole genome CRISPR-Cas9 screening approach to elucidate host molecules required for β-CoV infection and inflammatory cell death, PANoptosis, in macrophages, a sentinel innate immune cell. Our screen was validated through the identification of the known MHV receptor *Ceacam1* as the top hit, and its deletion significantly reduced viral replication due to loss of viral entry, resulting in a downstream reduction in MHV-induced cell death. Moreover, this screen identified several other host factors required for MHV infection-induced macrophage cell death. Overall, these findings demonstrate the feasibility and power of using genome-wide PANoptosis screens in macrophage cell lines to accelerate the discovery of key host factors in innate immune processes and suggest new targets for therapeutic development to prevent β-CoV-induced pathology.

## Abstract (web summary)

A CRISPR screen identified host factors that promote viral entry and inflammatory cell death, PANoptosis, upon infection with the murine hepatitis virus, a type of beta-coronavirus, suggesting potential therapeutic targets for coronavirus infections.

## Introduction

Coronavirus infections are among the current leading causes of global morbidity and mortality. The global burden of confirmed COVID-19 cases has surpassed 775 million, resulting in more than 6.9 million deaths as of May 22, 2023^1^. COVID-19 is caused by the β-coronavirus (β-CoV) severe acute respiratory syndrome coronavirus 2 (SARS-CoV-2)^2^. β-CoVs are positive sense single-stranded RNA (ssRNA) viruses that carry a relatively large RNA genome of about 30 kb^3,4^. These viruses cause respiratory infections that result in severe inflammatory immune pathology that drives morbidity and mortality^5^. Newly developed vaccines have significantly reduced symptomatic COVID-19 infections and mortality^6^. However, the progress in developing therapeutics to treat these infections is lagging behind, and there remains an urgent unmet need to provide treatment for patients and prevent severe disease manifestations.

Identifying new treatment strategies requires a mechanistic understanding of viral entry and innate immune activation to identify therapeutic targets. Innate immunity generally provides the first line of defense against infection and disease, but in the case of β-CoV infections, excess innate immune activation has been associated with the release of high levels of pro-inflammatory cytokines and chemokines, leading to a cytokine storm and pathogenesis^7–12^. Exacerbated cytokine release and organ failure upon infection with respiratory RNA viruses can be caused by aberrant innate immune-mediated regulated cell death^13–18^. Cell death pathways can be characterized as non-lytic (e.g., apoptosis) and lytic (e.g., pyroptosis and necroptosis). Mechanistically, apoptosis is initiated by caspase-8 and -9 and executed by caspase-3 and -7^19^; pyroptosis is induced by inflammatory caspases, caspase-1 and caspase-11 (mouse)/caspase-4 and -5 (human), and executed by gasdermin family members^20–24^; and caspase-8 inhibition leads to necroptosis via the RIPK3/MLKL pathway^25–29^. Growing evidence has shown extensive crosstalk among these cell death pathways, leading to the identification of PANoptosis. PANoptosis is a unique innate immune lytic, inflammatory cell death pathway that is driven by caspases and RIPKs and regulated by multiprotein PANoptosome complexes. PANoptosomes form upon cytosolic pattern recognition receptor sensing of pathogens, pathogen-associated molecular patterns (PAMPs), damage-associated molecular patterns (DAMPs), or the cytokines produced downstream^30–35^.

PANoptosis has been extensively implicated in β-CoV pathology. For instance, mouse hepatitis virus (MHV), a prototypical β-CoV that mimics many of the key aspects of human β-CoV biology, induces NLRP3 inflammasome activation and PANoptosis in murine bone marrow-derived macrophages (BMDMs)^36^. Additionally, SARS-CoV-2 and MHV induce PANoptosis in response to IFN therapy in both human and mouse cells, and this cell death drives an inflammatory immune response and lethality in murine models^15^. Furthermore, TNF and IFN-γ released during SARS-CoV-2 infection induce robust PANoptosis that drives inflammation, cytokine storm, and morbidity and mortality in murine models^16^. Acute respiratory distress syndrome (ARDS), a determining feature of COVID-19 disease severity, is promoted by myeloid cell death in patients with COVID-19 and in the MHV mouse model^10,15^.

Given the clear connections between innate immune inflammatory cell death, PANoptosis, and pathology in β-CoV infections, it is critical to understand the molecular mechanisms involved in this pathway to identify new therapeutic targets. We therefore utilized a whole genome CRISPR-based knockout screen to identify the key host factors driving cell death in response to MHV infection. We identified Ceacam1 as a critical molecule that regulates viral infection and acts as an important node in inflammatory cell death. Our CRISPR screen also identified several other molecules that are likely to have critical roles in β-CoV-mediated cell death that require further evaluation in future studies. Blocking these mechanisms presents a promising strategy to inhibit inflammatory cell death and reduce pathology in β-CoV infections.

## Results

### Genome-wide CRISPR screen identified host factors required for MHV-induced cell death

While extensive connections have been found between innate immune inflammatory cell death, PANoptosis, and pathology in β-CoV infections^15,16,36^, a need for a comprehensive understanding of the host cellular pathways involved in β-CoV infections remains to identify additional therapeutic targets. Therefore, to identify the host factors that are essential for β-CoV infection-induced cell death, we performed a whole genome CRISPR screen using the prototypical β-CoV MHV as a model. To develop the screen, we first prepared iBMDMs from Cas9-transgenic mice. These iBMDMs have several advantages, including their ease of use and nearly unlimited supply, and they represent a highly susceptible cell type with intact protein machinery to induce innate immune and cell death pathways.

**Fig. 1:**
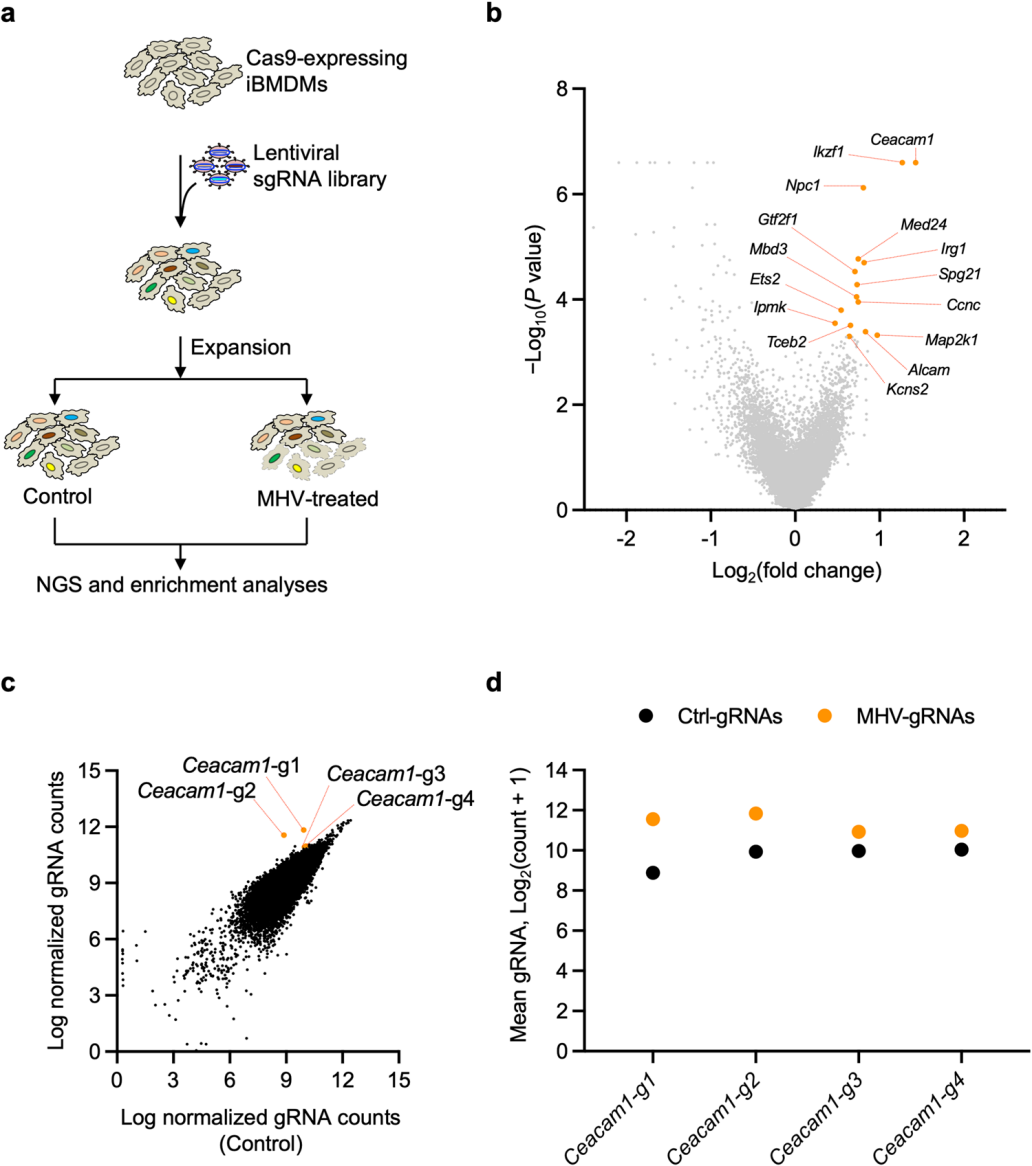
CRISPR screen identifies host factors required for β-CoV-induced cell death. **a** Schematic of the CRISPR screen workflow in mouse hepatitis virus (MHV)-infected immortalized bone marrow-derived macrophages (iBMDMs). **b** Representative volcano plot showing the log_2_ mean fold change for the gRNAs in the CRISPR screen following infection of iBMDMs carrying gRNAs with MHV (MOI 0.1) for 24 h. The top 15 CRISPR screen hits are labeled. **c** Scatter plot highlighting the enrichment of all four gRNAs targeting *Ceacam1* in the pool of iBMDMs carrying gRNAs from the whole genome CRISPR screen following infection with MHV (MOI 0.1) for 24 h. **d** Scatter plot depicting the distribution of the normalized gRNA counts in log scale for the individual *Ceacam1* gRNAs, represented in panels (**b**) and (**c**). Ctrl-gRNAs denotes the count in the control pool of cells that were not infected; MHV-gRNAs denotes the count in the pool of cells following infection with MHV (MOI 0.1) for 24 h.

To perform the CRISPR screen, we used the whole genome mouse pooled Brie library (Addgene, 73633), containing 78,637 individual guides targeting a total of 19,674 genes, with an average of 4 guides for each gene, as well as 1000 control non-targeting gRNAs^37^. We generated a pool of iBMDMs with individual genes deleted by CRISPR and infected these with MHV (MOI = 0.1) for 24 h. The surviving cells were analyzed to identify gRNAs that were enriched, suggesting that these genes have a role in inducing cell death in response to MHV infection (Fig. 1a). The screen identified several host genes that are potentially involved in MHV-induced cell death (pro-death host factors) (Fig. 1b). Analysis of the enriched genes indicated that gene sets corresponding to multiple biological processes and cellular components were significantly enriched among the pro-death host genes (Supplementary Fig. 1a, b). Several of the enriched gene sets encompass the host molecular machinery required for RNA transcription, amino acid metabolism, and the antiviral interferon signaling cascades (Supplementary Fig. 1a). Moreover, GO term analyses focused on the cellular components identified that most of the enriched gRNAs were regulatory genes of the endo-lysosomal compartment and mediators of RNA polymerase-II-dependent transcription (Supplementary Fig. 1b); these are also known to be important for both viral replication and intracellular maturation of the β-CoV particles^38–42^.

We next focused on the most highly enriched individual gRNAs and found that these corresponded to the MHV viral receptor carcinoembryonic antigen-related cell adhesion molecule 1 (*Ceacam1*) (Fig. 1b). Ceacam1 is known to be a critical host factor that dictates the cell tropism of MHV^43^, providing validation for the specificity of the findings from the CRISPR screen. Moreover, individual gRNA analyses showed uniform enrichment of all four unique gRNAs targeting *Ceacam1*, confirming that gRNAs for *Ceacam1* are among the most positively enriched genes in the MHV CRISPR screen (Fig. 1c, d). Together, these findings suggest that a whole genome CRISPR screen can identify host molecules that are essential for β-CoV–mediated cellular effects.

### MHV infection induces Ceacam1-dependent inflammatory cell death, PANoptosis, and cytokine release in iBMDMs

**Fig. 2:**
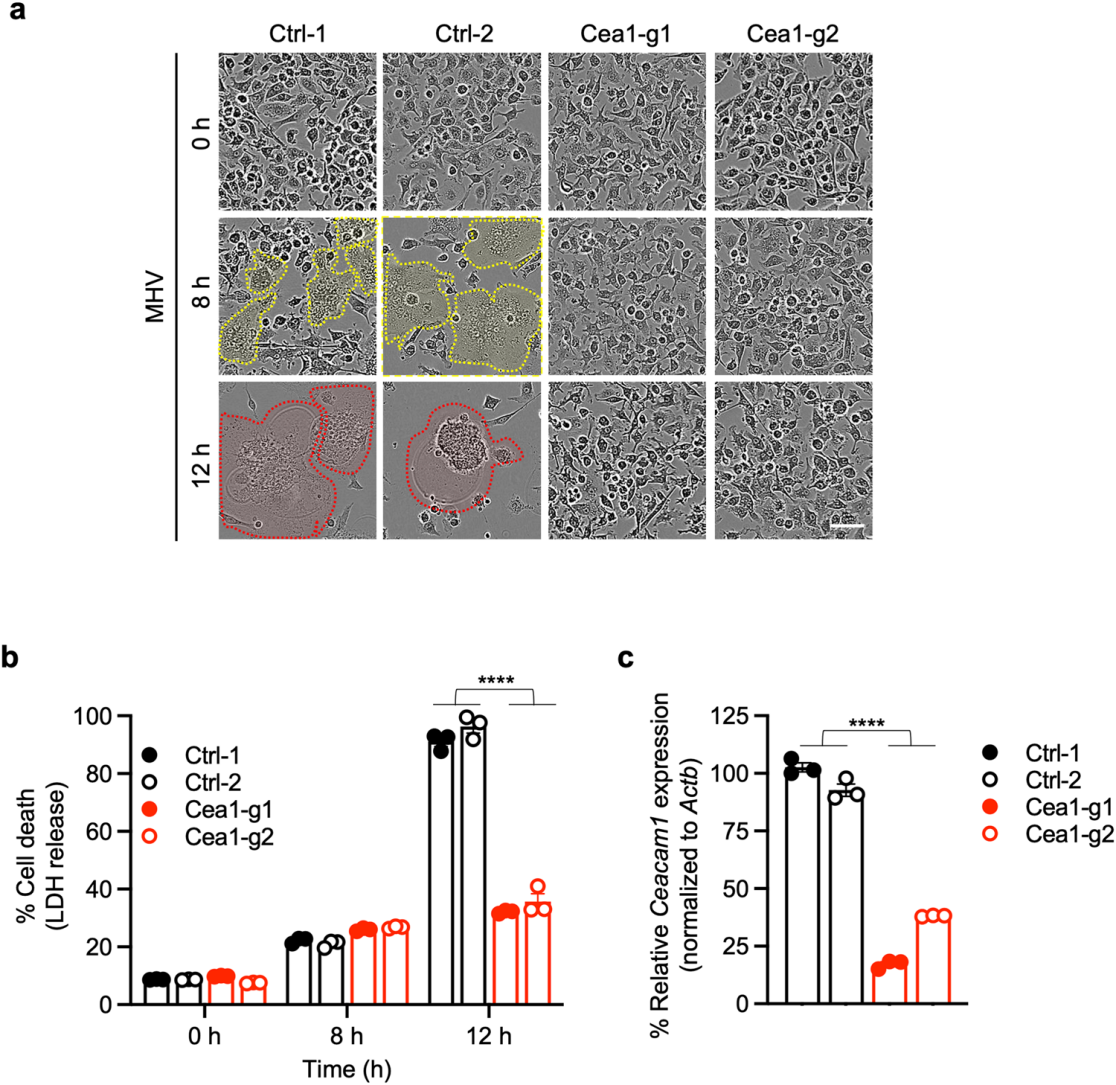
Ceacam1 is required for MHV-induced cell death. **a** Cell death analysis in mouse hepatitis virus (MHV; MOI 0.1)-infected immortalized bone marrow-derived macrophages (iBMDMs) with and without *Ceacam1* gRNA treatment with two different guides (Cea1-g1 and Cea1-g2). The yellow dotted lines denote syncytia, and the red dotted lines denote ballooning and dying syncytia. **b** Percentage of cell death at indicated time points after MHV infection in iBMDMs with and without *Ceacam1* gRNA treatment in terms of LDH release. **c** Knockdown efficiency of the two different *Caecam1* gRNAs, Cea1-g1 and Cea1-g2, employed in this study. *Actb* was used to normalize the *Caecam1* expression. The data presented are representative of three independent experiments (**a**–**c**). Data are shown as mean ± SEM (**b**, **c**). Analysis was performed using the Student’s t-test; ****P < 0.0001. Ctrl: Control with no gRNA. The scale bar is representative of 50 μm.

We next sought to validate the CRISPR screen findings using *Ceacam1* as the lead candidate gene. To this end, we first assessed the kinetics of cell death in MHV-infected iBMDMs. We observed robust cell death in MHV-infected wild-type (WT) iBMDMs that increased over time (Fig. 2a, b), while gRNA-mediated deletion of *Ceacam1* significantly protected the iBMDMs from cell death (Fig. 2a–c). Moreover, the characteristic syncytia formation mediated by the virus spike protein during MHV infection was observed in WT iBMDMs (Fig. 2a), similar to other cell types^44,45^, but not in cells with gRNA-mediated deletion of *Ceacam1* (Fig. 2a, c).

**Fig. 3:**
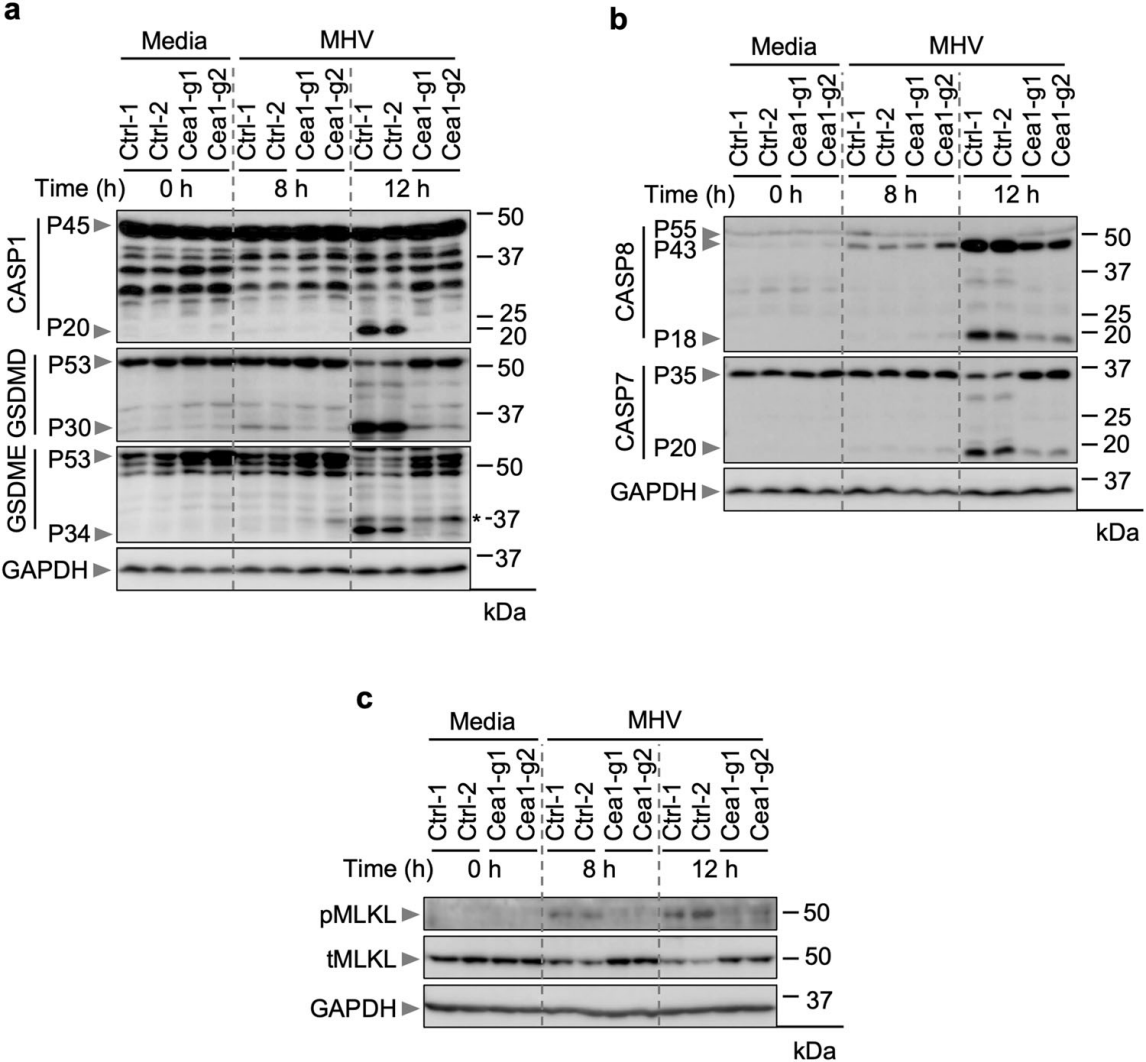
Ceacam1 modulates MHV-induced PANoptosis. **a**–**c** Immunoblot analysis of pro- (P45) and cleaved caspase-1 (P20; CASP1), pro- (P53) and activated (P30) gasdermin D (GSDMD), and pro- (P53) and activated (P34) gasdermin E (GSDME) (**a**); pro- (P55) and cleaved caspase-8 (P43, P18; CASP8) and pro- (P35) and cleaved caspase-7 (P20; CASP7) (**b**); and phospho- (pMLKL) and total MLKL (tMLKL) (**c**) from mouse hepatitis virus (MHV; MOI 0.1)-infected immortalized bone marrow-derived macrophages (iBMDMs) with and without *Ceacam1* gRNA treatment with two different guides (Cea1-g1 and Cea1-g2) at the indicated time points. Asterisk denotes a non-specific band. Blots were reprobed for GAPDH to serve as the internal control. The data presented are representative of three independent experiments (**a**–**c**). Ctrl: Control with no gRNA.

Previous studies showed that MHV infection in macrophages induces PANoptosis, characterized by the activation of multiple caspases and cell death markers^15,36^. These include caspase-1 to promote the processing of GSDMD to its P30 N-terminal fragment (GSDMD-N)^15,36^, which forms pores in the cell membrane to trigger cell death^20–22,46,47^. We therefore sought to determine whether the same is true in iBMDMs and understand how the loss of Ceacam1 affects these molecular processes. We observed the cleaved, active form of caspase-1 in WT iBMDMs after MHV infection (Fig. 3a), indicating the occurrence of inflammasome activation. Additionally, we observed the P30 fragment of GSDMD and the P34 fragment of GSDME (Fig. 3a). Consistent with the cell death data (Fig. 2a, b), gRNA-based deletion of *Ceacam1* markedly reduced the activation of caspase-1 and GSDMD (Fig. 3a). Also, in line with previous reports in other cell types^48–51^, we observed activation of caspases-8 and -7 in WT iBMDMs in response to MHV infection (Fig. 3b). However, the gRNA-based deletion of *Ceacam1* reduced the cleavage and activation of these caspases (Fig. 3b). In addition, in WT iBMDMs, we detected phospho-MLKL (Fig. 3c), another pore-forming molecule known to induce cell death, and phospho-MLKL was markedly reduced in *Caecam1-*deficient iBMDMs (Fig. 3c). Furthermore, gRNA-mediated knockdown of *Ceacam1* reduced the release of pro-inflammatory cytokines IL-1β and KC at 12 h post-MHV infection (Supplementary Fig. 2a–c). Together, these observations confirm Ceacam1’s positive role in MHV-induced PANoptotic cell death and subsequent inflammatory cytokine release.

### Ceacam1 is required for MHV viral replication

**Fig. 4:**
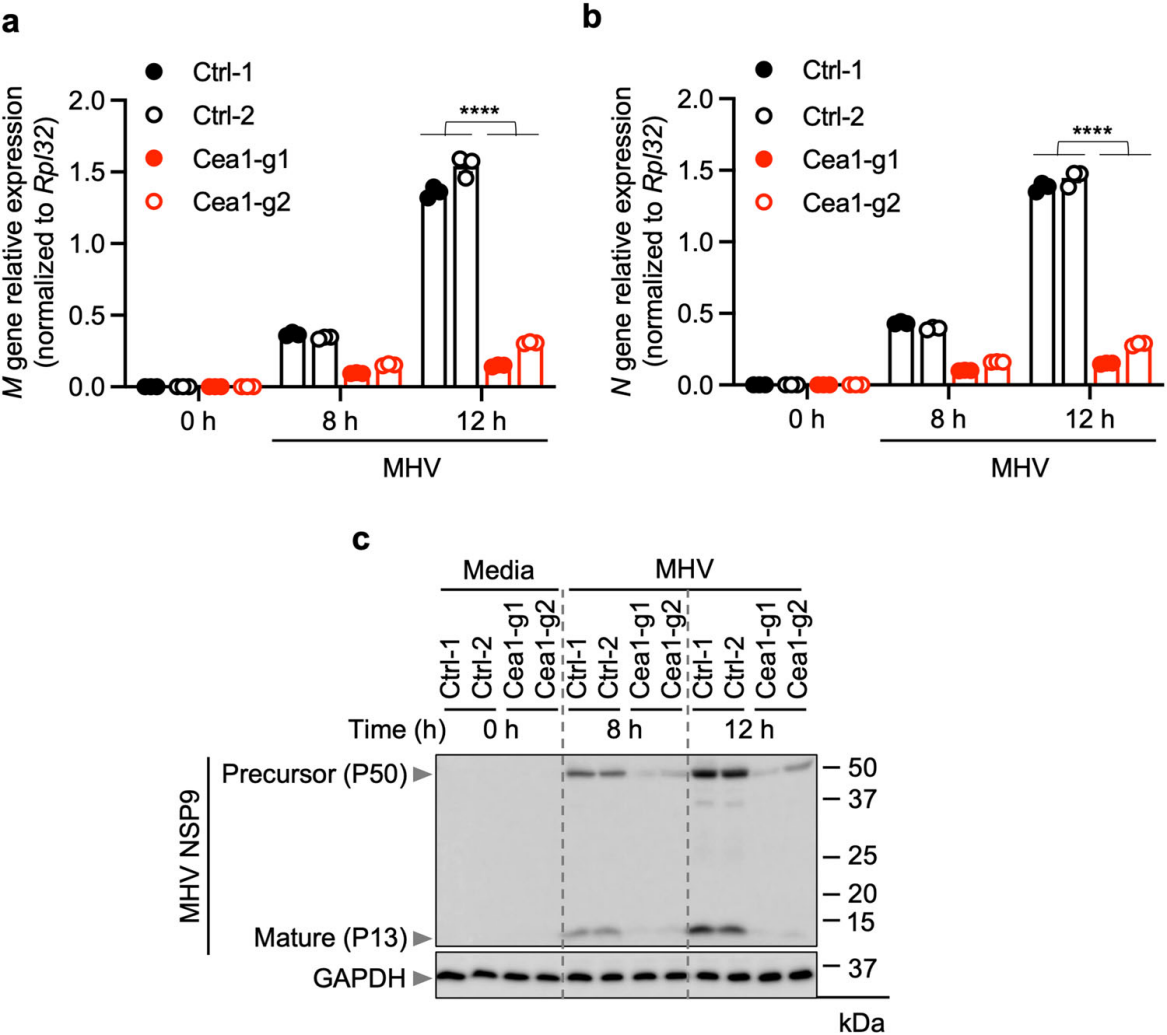
Loss of *Ceacam1* reduces MHV replication. **a**, **b** Quantification of mRNA levels of the mouse hepatitis virus (MHV) M (**a**) and N (**b**) structural proteins at the indicated time points after MHV infection (MOI 0.1) in immortalized bone marrow-derived macrophages (iBMDMs) with and without *Ceacam1* gRNA treatment with two different guides (Cea1-g1 and Cea1-g2). **c** Immunoblot analysis of non-structural protein 9 (NSP9) precursor (P50 and P37) and mature forms (P13) in iBMDMs following the indicated treatment. Blots were reprobed for GAPDH to serve as the internal control. The data presented are representative of three independent experiments (**a**–**c**). Data are shown as mean ± SEM (**a**, **b**). Analysis was performed using the Student’s t-test; ****P < 0.0001. Ctrl: Control with no gRNA.

During infection, inflammatory cell death is activated downstream of innate immune sensing of viral PAMPs and DAMPs^52^. Inhibition of viral replication may reduce the amounts of viral PAMPs and DAMPs present, thereby reducing cell death. Given the known function of Ceacam1 in viral entry^53,54^, we hypothesized that infectivity may be the mechanism by which loss of *Ceacam1* inhibits cell death. To test this hypothesis, we examined intracellular levels of viral nucleic acids and proteins from MHV as a measure of viral replication and viral PAMP abundance. We observed a substantial increase in the expression of MHV mRNA for the structural proteins M and N in WT iBMDMs, but not in *Ceacam1*-deficient cells (Fig. 4a, b). Moreover, we also observed the time-dependent production of the MHV non-structural protein NSP9 in both its pro- and cleaved/matured forms in WT iBMDMs, but not in *Ceacam1* gRNA-treated iBMDMs (Fig. 4c). Treatment of iBMDMs with GC376, an inhibitor of viral replication that blocks the activity of the β-CoV main protease (M^pro^), substantially reduced the MHV-infected cell death, PANoptosis (Supplementary Fig. 3a–c). Together, these data establish the Ceacam1 receptor as an important viral entry point for MHV, allowing the virus to enter the cytosol to replicate and induce subsequent inflammatory cell death in iBMDMs.

## Discussion

β-CoV-induced cell death pathways have been widely implicated in disease pathogenesis^5,6,8–10,15,16^. Our findings here show that the MHV-induced, *Ceacam1*-dependent cell death in myeloid iBMDMs is characterized by activation of caspases-1, -8, and -7, GSDMD, GSDME, and MLKL. These results suggest that Ceacam1 acts upstream of multiple cell death molecules by promoting the intracellular entry of MHV, which is required for replication of the virus to produce viral PAMPs and DAMPs that induce cell death activation. Moreover, we found that GC376 also inhibits MHV-induced inflammatory cell death, PANoptosis. GC376 is a highly potent dipeptidyl aldehyde bisulfite adduct inhibitor that acts as a prodrug and releases active GC373 inside the cells. Once in the cell, GC373 specifically binds to the active site cysteines of the M^pro^ of positive sense ssRNA viruses, such as β-CoVs, and blocks the proteolytic processing of the viral polyprotein to prevent viral replication^55,56^. Together, these observations establish a link between viral replication and the induction of inflammatory cell death in myeloid cells. While several studies have demonstrated that the replication of β-CoVs in myeloid cells in vivo is poor, this replication is also not essential for pathogenesis. Myeloid cells can phagocytose or sense the DAMPs and PAMPs from infected and dying epithelial and other non-immune cells around them and further amplify the inflammatory mechanisms of cell death and pathophysiology^16,57–59^, highlighting the importance of understanding the induction of cell death in this population. Furthermore, our study also emphasizes the utility of using whole genome CRISPR screening technology to identify new cellular processes in innate immune myeloid cellular models of viral infections. Using this technology enables the evaluation of both known and unknown cell death mediators in parallel to discover new regulatory mechanisms. Our CRISPR screen identified several hits that are likely to be involved in regulating β-CoV-induced cell death, and these hits can be further assessed in future studies to characterize therapeutic targets.

MHV is known to mimic many aspects of the pathology and pathogenesis of the human β-CoVs, including SARS-CoV-2^54,60^. For example, MHV can induce cytokine storm and syncytia formation, which are important predictive features of mortality in cases of SARS-CoV-2 infection^61–63^. Syncytia formation is thought to promote viral spread to neighboring cells, which can drive cell death, and syncytia are frequently observed in patients with severe COVID-19^64,65^. However, MHV does not bind to human CEACAM1 and hence cannot infect human cells^53,54^. Nonetheless, the many similarities between MHV and SARS-CoV-2 viral replication, pathogenic mechanisms, and the availability of well-established mouse models have helped the field substantially further our understanding of SARS-CoV-2-related pathologies, highlighting the continued utility of this model.

Overall, our study leveraged a whole genome CRISPR screen approach to identify the host cellular receptor Ceacam1 as a key regulator of MHV-mediated cell death, demonstrating the feasibility and power of using genome-wide screens in macrophage cell lines to accelerate the discovery of key host factors in innate immune processes. Moreover, our study also supports the potential of using blocking antibodies or targeted degradation of receptors such as Ceacam1 to block viral entry and the associated pathologies. Indeed, comparable approaches to block viral entry and replication are being employed and tested successfully in preclinical and clinical studies in a variety of viral infections, both in animal models and in clinical trials^66,67^. Understanding the molecular mechanisms of cell death in β-CoV infections provides new insights for the development of novel therapeutic and prophylactic strategies to counteract current and emerging pathogens and prevent pathology.

## Methods

### Generation of Brie lentiviral library

The Mouse Brie CRISPR KO library was a gift from David Root and John Doench (Addgene #73632 and #73633). The plasmid library was amplified and validated in the Center for Advanced Genome Engineering at St. Jude Children’s Research Hospital (St. Jude) as described in the Broad GPP protocol, with the only exception being the use of Endura DUOs electrocompetent cells. The St. Jude Hartwell Center Genome Sequencing Facility provided all NGS sequencing. Single-end 100 cycle sequencing was performed on a NovaSeq 6000 (Illumina). Validation to check gRNA presence and representation was performed using calc_auc_v1.1.py (https://github.com/mhegde/) and count_spacers.py^68^. Viral particles were produced by the St. Jude Vector Development and Production Laboratory. CRISPR KO screens were analyzed using Mageck-Vispr v0.5.7^69^.

### Mouse hepatitis virus (MHV) culture

The mouse hepatitis virus (A59 strain) was propagated in 17Cl-1 cells as previously described^70^. The virus titer was measured by plaque assay in 17Cl-1 cells.

### CRISPR screen

Cas9-expressing immortalized bone marrow-derived macrophages (iBMDMs) were generated from Cas9-GFP knock-in mice (Jackson Laboratories, 026179). All work with animals was reviewed and approved by the St. Jude Children’s Research Hospital IACUC. The iBMDMs were infected with lentiviral particles at an MOI of 0.3 to ensure that the majority of the iBMDMs received a single gRNA per cell to generate a pool of individual knockout cells. Two replicates of an adequate number of cells were used as controls to obtain a representation (screen depth) of >500 cells for each sgRNA in the library, and a similar number of cells from the same batch of virus preparation were infected with MHV at a multiplicity of infection (MOI) of 0.1 for 24 h. The uninfected control cell population and the surviving cells from the MHV-infected samples were subjected to CRISPR screen enrichment analysis. Total genomic DNA was isolated using NucleoSpin® Blood kits (Takara Bio Inc., USA; 740954 and 740950), and the concentrations of the isolated gDNA samples were measured using NanoDrop (Thermo Fisher Scientific, USA).

The MAGeCK pipeline was used to estimate the log_2_ (fold change) with significance levels for each gene in the CRISPR screen. Genes with positive fold change were expected to be required for cell death. The top gene hits along with their significance from the CRISPR screen were visualized using a volcano plot and an RRA score plot using MAGeCKFlute v2.0.0^71^. The expression profiles of individual gRNAs for control and treatment samples were visualized using a scatter plot. The gene ontology (GO)-term analyses of biological processes and cellular components of the significantly enriched genes from the upregulated list of gRNAs in the MHV whole genome CRISPR screen were analyzed using the widely used web-based application package, Enrichr^72–74^, which integrates gene-set libraries and ranks the gene sets based on the enrichment scores of the given set of genes (https://maayanlab.cloud/Enrichr/).

### Cas9-iBMDM culture and infection

Cas9-expressing iBMDMs were electroporated separately with gRNAs for *Ceacam1* (sequences: Ceacam1.gRNA1 (Cea1-g1): 5’-ATAGTAATATGAATTTCACG-3’; Ceacam1.gRNA2 (Cea1-g2): 5’-TTGTTGTCTTCAGCAACCTG-3’). The gRNA electroporated cells were allowed to rest for 5 days to achieve CRISPR-based deletion of the targeted genes. The cells were then expanded and seeded in 12- and 24-well plates at a seeding density of 1 × 10^6^ cells/mL and 0.5 × 10^6^ cells/mL, respectively, in complete DMEM (DMEM supplemented with 10% heat-inactivated fetal bovine serum (HI-FBS; S1620, Biowest) and 1% penicillin-streptomycin (15070-063, Thermo Fisher Scientific)), and rested for about 24 h before the experiments. The cells were then washed with PBS and infected with MHV at an MOI of 0.1 in DMEM plain media (Sigma, D6171) with 1% penicillin/streptomycin (Thermo Fisher Scientific, 15070-063). After 1 h incubation with MHV, cells were supplemented with FBS to a final concentration of 10% and used for IncuCyte imaging or incubated for the indicated times before collecting samples for downstream analyses. Where indicated, the iBMDMs were treated with GC376 (Cayman Chemical, #31469), starting at 1 h post-infection with MHV, using the indicated concentrations.

### Cell death analysis

The IncuCyte^®^ S3 and/or SX5 Live-Cell Analysis System was used to image and analyze cell death in real-time, as previously described^75^. In brief, Cas9-iBMDMs electroporated with non-targeting and *Ceacam1* gRNA were seeded in 24-well plates (0.5 × 10^6^ cells/well) and infected with MHV as described above. After 1 h of incubation, propidium iodide (PI; Life Technologies, P3566) was added to the cells together with FBS. The plate was scanned for phase-contrast images (4 image fields/well) in real-time for 24 h at an interval of 1 h. Cell death was quantified in terms of percentage LDH release and assessed using the Cyto Tox 96® Non-Radioactive Cytotoxicity assay kit (Promega G1782) according to the manufacturer’s instructions.

### ELISA

Cytokines IL-1β, KC, and IL-6 were quantified according to the manufacturer’s instructions using the MILLIPLEX® MAP Mouse cytokine/chemokine bead panel (Millipore, MCYTOMAG-70K). The samples were analyzed using the MILLIPLEX Analyzer with the *xPONENT* software.

### Western blotting

To blot for caspases, Cas9-iBMDMs were lysed along with the supernatant using 125 μL 4× SDS loading buffer, containing 50 μL of caspase lysis buffer (1× protease inhibitors, 1× phosphatase inhibitors, 10% NP-40, and 25 mM DTT). To detect other proteins in the cell lysates alone, the supernatants were aspirated, and the cells were lysed using RIPA buffer. Caspase and RIPA lysates were separated on 10–12% polyacrylamide gels, then transferred onto PVDF membranes. After blocking the membranes with 5% skim milk for 1 h at room temperature, the blots were incubated with their respective primary antibodies overnight at 4 °C. Subsequently, the blots were washed in 1× TBST and incubated with secondary antibodies conjugated with HRP for 1 h at room temperature. The GE Amersham Imager 600 was used to develop the blots. The antibodies used were as follows: anti-caspase-1 (AdipoGen, AG-20B-0042, 1:1000), anti-caspase-7 (CST, #9492, 1:1000), anti-cleaved caspase-7 (CST, #9491, 1:1000), anti-caspase-8 (CST, #4927, 1:1000), anti-cleaved caspase-8 (CST, #8592, 1:1000), anti-GSDMD (Abcam, ab209845, 1:1000), anti-GSDME (Abcam, AB215191, 1:1000), anti-pMLKL (CST, #37333, 1:1000), anti-tMLKL (Abcepta, AP14272B, 1:1000), GAPDH (CST, #5174S, 1:5000), NSP9 of MHV (Rockland Immunochemicals, 200-301-A56, 1:1000), and HRP-conjugated secondary antibodies (Jackson ImmunoResearch Laboratories, anti-rabbit [111-035-047], 1:5000; anti-mouse [315-035-047], 1:5000).

### Quantitative PCR

Total RNA was isolated using TRIzol^®^ reagent (Ambion, #15596018). A total of 500 ng of RNA was reverse transcribed to cDNA using the High-capacity cDNA synthesis reverse transcription kit (Applied Biosystems, #4368813), as per the manufacturer’s instructions. The cDNA was used for measurement of *Ceacam1*, viral *M* gene, and viral *N* gene expression in MHV-infected Cas9-expressing iBMDMs with or without *Ceacam1* depletion by gRNA treatment (described above). mRNA levels were quantified in real time using Sybr^®^ Green (Applied Biosystems, #4367659) and the Quant Studio™ 7 Flex Real-Time PCR System (Applied Biosystems). The expression levels were normalized to *Rpl32* in the case of the viral genes and *Actb* for the host cellular *Ceacam1* gene expression and presented as fold change. Forward and reverse primer sequences used in the study are as follows: *M* gene, Forward: 5-GGAACTTCTCGTTGGGCATTATACT-3, Reverse: 5-ACCACAAGATTATCATTTTCACAACATA-3; *N* gene, Forward: 5-CAGATCCTTGATGATGGC GTAGT-3, Reverse: 5- AGAGTGTCCTATCCCGACTTTCTC-3; *Rpl32*, Forward: 5-AAGCGAAA CTGGCGGAAAC-3, Reverse: 5-TAACCGATGTTGGGCATCAG-3; *Ceacam1*, Forward: 5-ATTT CACGGGGCAAGCATACA-3, Reverse: 5-GTCACCCTCCACGGGATTG-3; and *Actb*, Forward: 5-CAGCTTCTTTGCAGCTCCTT-3, Reverse: 5-CACGATGGAGGGGAATACAG-3.

### Statistics and reproducibility

Data analysis was performed using GraphPad Prism 7.0 software. Data are presented as mean ± SEM from three independent repeats. The student’s *t* test was used to determine the statistical significance. *P* values ≤0.05 were considered significant, where **P < 0.01, ***P < 0.001, and ****P < 0.0001.

### Reporting summary

Further information on research design is available in the Nature Portfolio Reporting Summary linked to this article.

### Supplementary information

Supplementary Information

Description of Additional Supplementary Files

Supplementary Data 1

Reporting Summary

## Data Availability

Next-generation sequencing results from the CRISPR screen are deposited in BioProject: PRJNA1009133. All other datasets are included in the published article and the supplementary information. The uncropped western blots are included in supplementary information (Supplementary Figs. 4–6). The numerical data points for the graphs are provided in Supplementary Data 1. All other data are available from the corresponding authors upon reasonable request.

## Code availability

This paper does not report original code.
