# Supplementary material for: Inflammatory cell death, PANoptosis, screen identifies host factors in coronavirus innate immune response as therapeutic targets
Source: Commun Biol. 2023 Oct 20;6:1071. doi: 10.1038/s42003-023-05414-9 (PMC10589293; doi:10.1038/s42003-023-05414-9)
Supplement: Supplementary file 1 — Supplementary Information [file 42003_2023_5414_MOESM1_ESM.pdf]

Supplementary Information:

Inflammatory cell death, PANoptosis, screen identifies host factors in coronavirus innate immune response as therapeutic targets

R.K. Subbarao Malireddi[1#], Ratnakar R. Bynigeri[1#], Raghvendra Mall[1,2], Jon P. Connelly[3], Shondra M. Pruett-Miller[3], Thirumala-Devi Kanneganti[1]*

[1]Department of Immunology, St. Jude Children's Research Hospital, Memphis, TN, 38105, USA

[2]Current affiliation: Biotechnology Research Center, Technology Innovation Institute, Abu Dhabi, P.O. Box 9639, United Arab Emirates

[3]Center for Advanced Genome Engineering (CAGE), St. Jude Children's Research Hospital, Memphis, TN 38105, USA

[#] These authors contributed equally to this work.

*To whom correspondence should be addressed:

Thirumala-Devi Kanneganti, Department of Immunology

St. Jude Children's Research Hospital, MS #351

262 Danny Thomas Pl, Memphis, TN 38105

Thirumala-Devi.Kanneganti@StJude.org

Tel: (901) 595-3634; Fax: (901) 595-5766

Keywords: innate immunity, cell death, coronavirus, murine hepatitis virus, gasdermin D, caspase-1, pyroptosis, gasdermin E, caspase-8, caspase-7, apoptosis, necroptosis, PANoptosis, PANoptosome, Ceacam1, CRISPR, inflammation, inflammatory, infection

Contents:

Supplementary figures: Supplementary Figures 1–6.

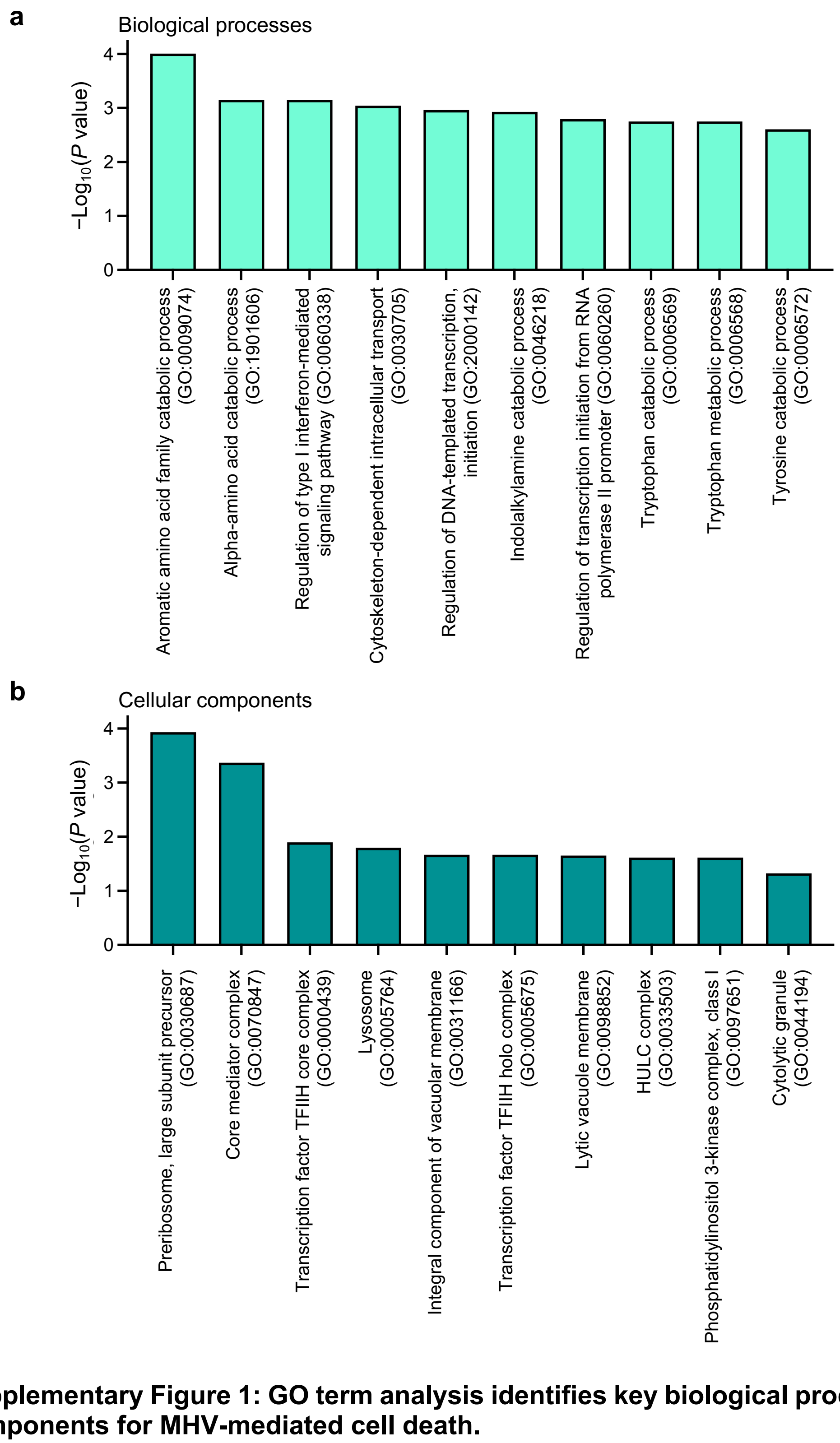

Supplementary Figure 1: GO term analysis identifies key biological processes and cellular components for MHV-mediated cell death.

a, b) The GO term analyses of biological processes (**a**) and cellular components (**b**) of the gene sets that were significantly enriched among the gRNAs in the mouse hepatitis virus (MHV) whole genome CRISPR screen using the Enrichr web-based tool (https://maayanlab.cloud/Enrichr/). The *P* values, **P* < 0.05, are considered statistically significant and included in the graphs.

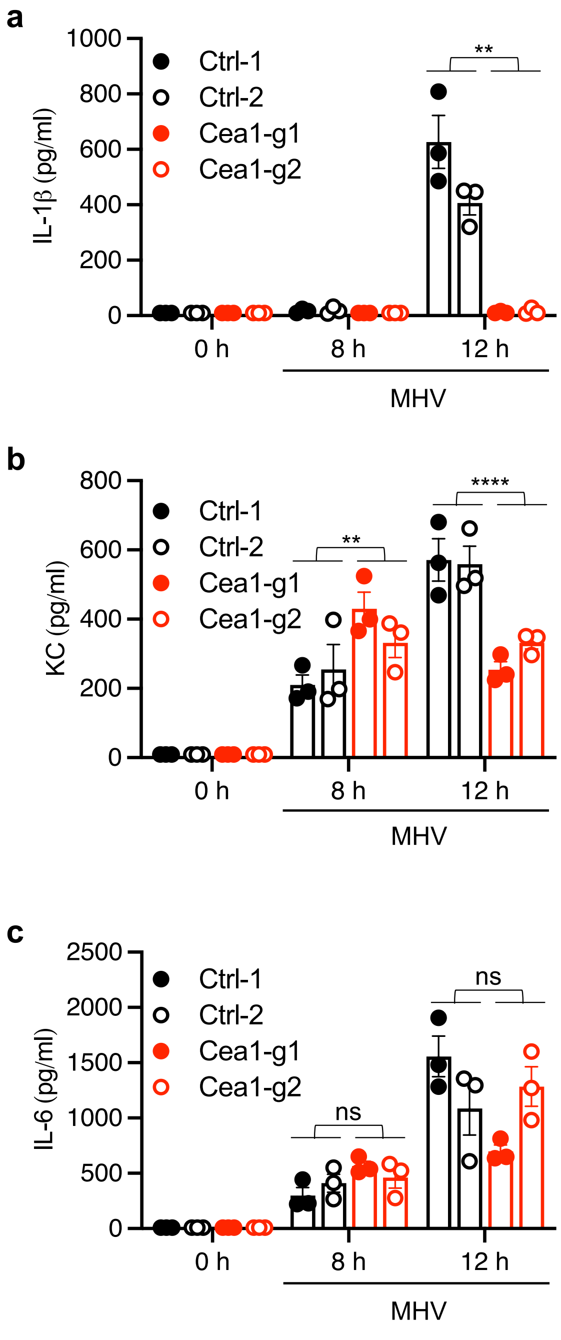

Supplementary Figure 2: Loss of *Ceacam1* blocks MHV-mediated IL-1β and KC release.
a–c) Cytokine release from mouse hepatitis virus (MHV; MOI 0.1)-infected immortalized bone marrow-derived macrophages (iBMDMs) with and without *Ceacam1* gRNA treatment with two different guides (Cea1-g1 and Cea1-g2) at the indicated timepoints. Data are shown as mean ± SEM and are presented from three biological replicates (a–c). Analysis was performed using the Student's t-test; ns, not significant, $^{**}P < 0.01$, $^{****}P < 0.0001$. Ctrl: Control with no gRNA.

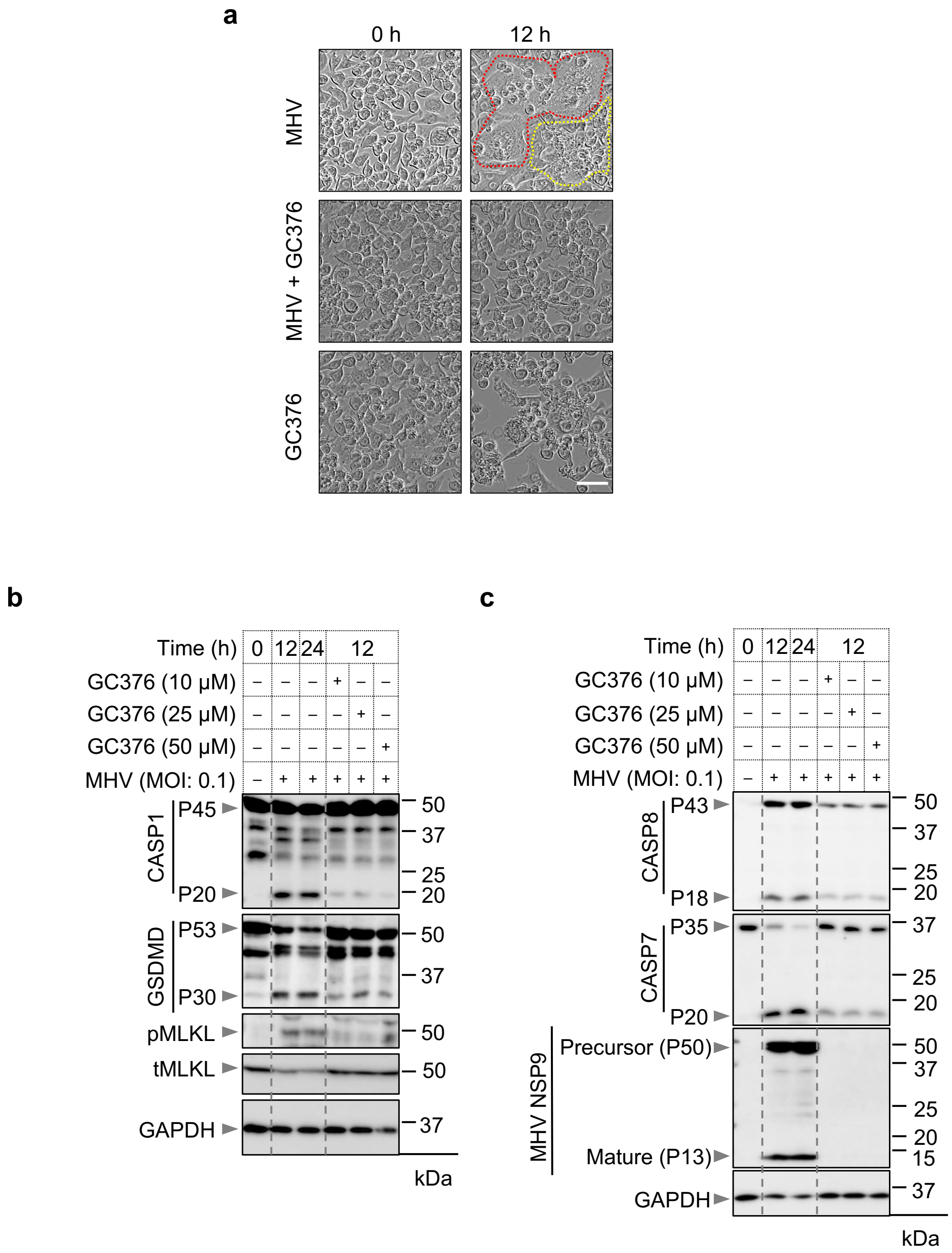

Supplementary Figure 3: The viral replication inhibitor GC376 blocks MHV-induced PANoptosis.

a) Cell death analysis in mouse hepatitis virus (MHV; MOI 0.1)-infected immortalized bone marrow-derived macrophages (iBMDMs) with or without treatment with GC376, an inhibitor of viral replication that blocks the activity of the β-CoV main protease (M^{pro}). The yellow dotted line denotes syncytia, and the red dotted line denotes ballooning and dying syncytia. **b–c**) Immunoblot analysis of pro- (P45) and cleaved caspase-1 (P20; CASP1), pro- (P53) and activated (P30) gasdermin D (GSDMD), and phospho- (pMLKL) and total MLKL (tMLKL) (**b**); and cleaved caspase-8 (p43 and P18; CASP8), pro- (P35) and cleaved caspase-7 (P20; CASP7), and non-structural protein 9 (NSP9) precursor (P50 and P37) and mature forms (P13) in iBMDMs following the indicated treatments (**c**). GAPDH immunoblots were used as internal controls. The data presented are representative of two independent experiments (a–c). The scale bar is representative of 50 μm.

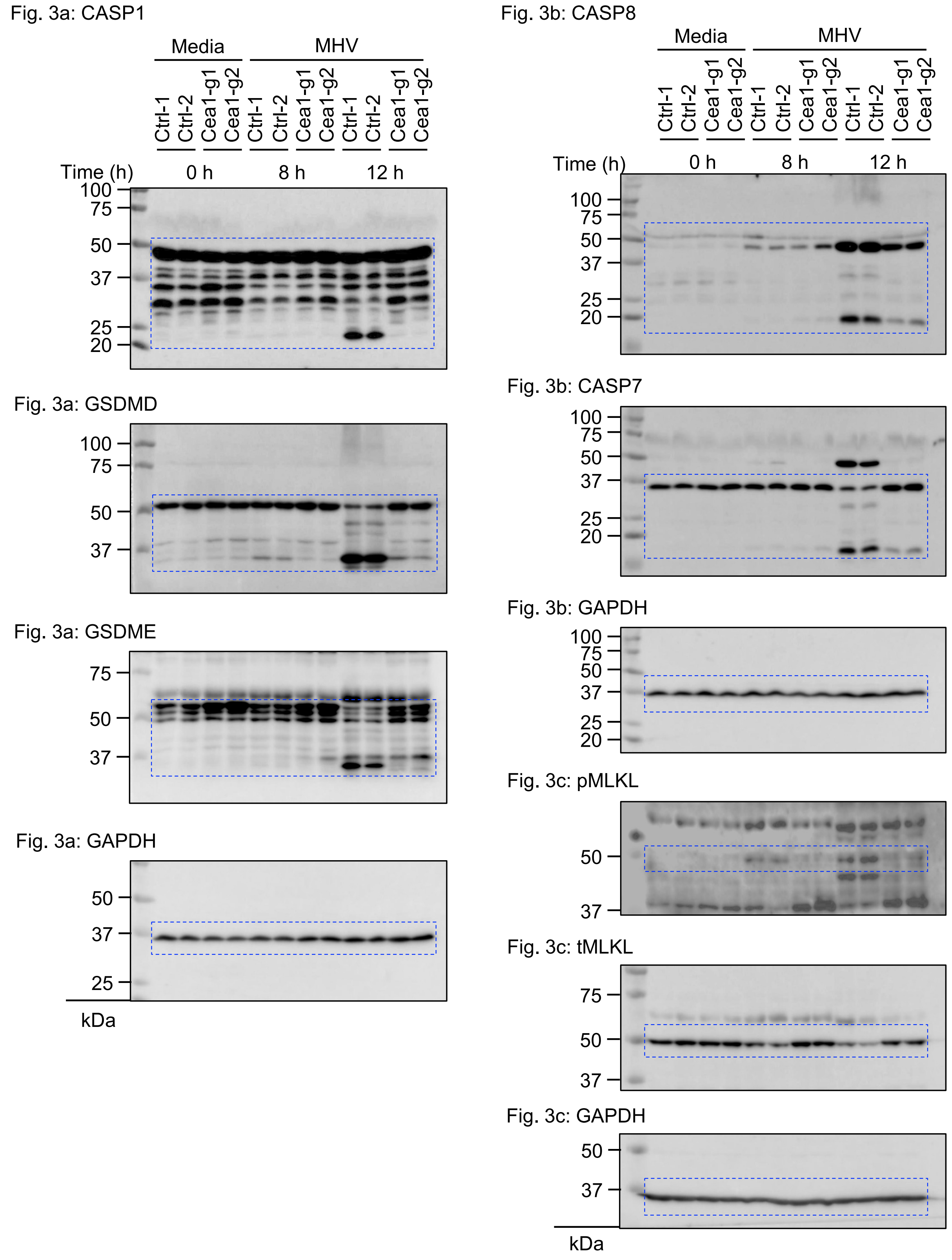

Supplementary Figure 4: Uncropped western blots for Figure 3.

Fig. 4c: MHV NSP9

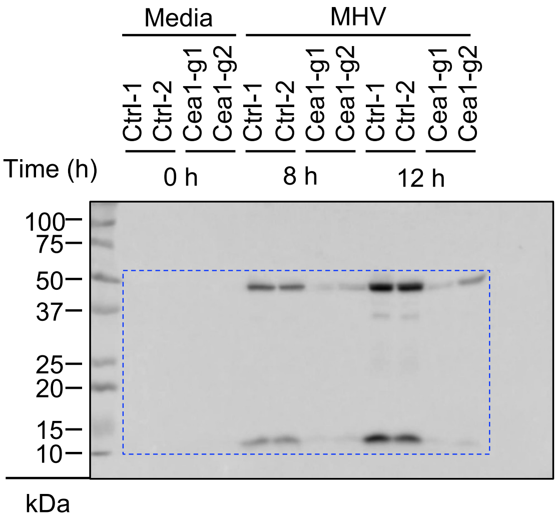

Fig. 4c: GAPDH

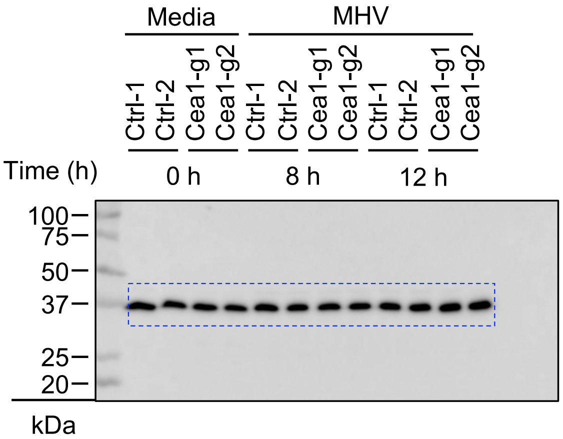

Supplementary Figure 5: Uncropped western blots for Figure 4.

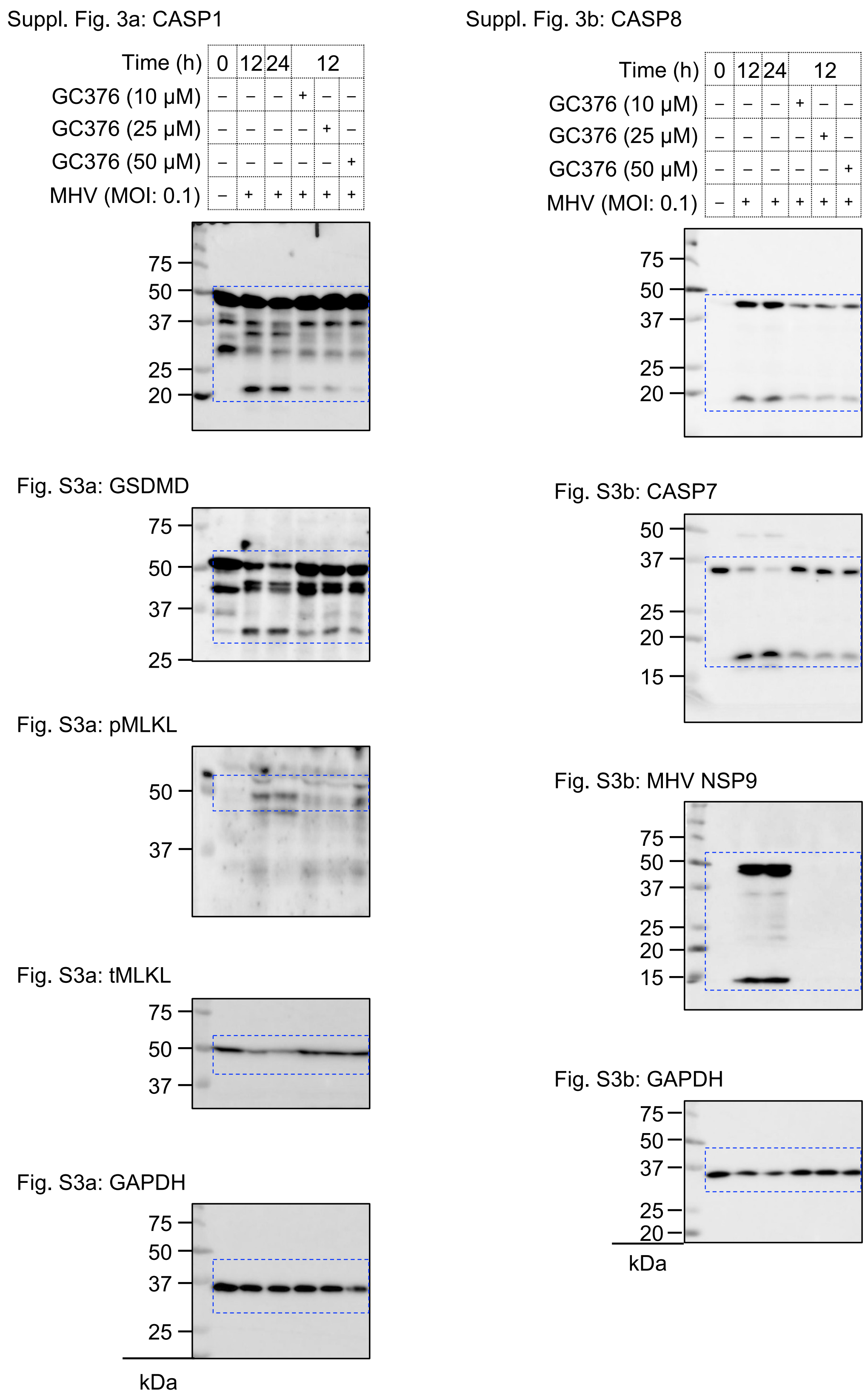

Supplementary Figure 6: Uncropped western blots for Supplementary Figure 3.
