# Supplementary material for: Inflammatory cell death, PANoptosis, screen identifies host factors in coronavirus innate immune response as therapeutic targets
Source: Commun Biol. 2023 Oct 20;6:1071. doi: 10.1038/s42003-023-05414-9 (PMC10589293; doi:10.1038/s42003-023-05414-9)
Supplement: Supplementary file 2 — Description of Additional Supplementary Files [file 42003_2023_5414_MOESM2_ESM.pdf]

Description of Additional Supplementary Files

File name: Supplementary Data 1
Description: The source data for graphs in the Figures and Supplementary Figures
