# Supplementary material for: Inflammatory cell death, PANoptosis, screen identifies host factors in coronavirus innate immune response as therapeutic targets
Source: Commun Biol. 2023 Oct 20;6:1071. doi: 10.1038/s42003-023-05414-9 (PMC10589293; doi:10.1038/s42003-023-05414-9)
Supplement: Supplementary file 4 — Reporting Summary [file 42003_2023_5414_MOESM4_ESM.pdf]

nature portfolio

Corresponding author(s): Thirumala-Devi Kanneganti

Last updated by author(s): Oct 11, 2023

Reporting Summary

Nature Portfolio wishes to improve the reproducibility of the work that we publish. This form provides structure for consistency and transparency in reporting. For further information on Nature Portfolio policies, see our Editorial Policies and the Editorial Policy Checklist.

Statistics

For all statistical analyses, confirm that the following items are present in the figure legend, table legend, main text, or Methods section.

n/a	Confirmed	
☐	☒	The exact sample size (*n*) for each experimental group/condition, given as a discrete number and unit of measurement
☐	☒	A statement on whether measurements were taken from distinct samples or whether the same sample was measured repeatedly
☐	☒	The statistical test(s) used AND whether they are one- or two-sided<br>*Only common tests should be described solely by name; describe more complex techniques in the Methods section.*
☒	☐	A description of all covariates tested
☐	☒	A description of any assumptions or corrections, such as tests of normality and adjustment for multiple comparisons
☐	☒	A full description of the statistical parameters including central tendency (e.g. means) or other basic estimates (e.g. regression coefficient) AND variation (e.g. standard deviation) or associated estimates of uncertainty (e.g. confidence intervals)
☐	☒	For null hypothesis testing, the test statistic (e.g. *F*, *t*, *r*) with confidence intervals, effect sizes, degrees of freedom and *P* value noted<br>*Give P values as exact values whenever suitable.*
☒	☐	For Bayesian analysis, information on the choice of priors and Markov chain Monte Carlo settings
☒	☐	For hierarchical and complex designs, identification of the appropriate level for tests and full reporting of outcomes
☒	☐	Estimates of effect sizes (e.g. Cohen's *d*, Pearson's *r*), indicating how they were calculated

Our web collection on statistics for biologists contains articles on many of the points above.

Software and code

Policy information about availability of computer code

Data collection	Microscopy images were collected using manufacturer supplied software (IncuCyte S3 and/or SX5). For immunoblotting, membranes were developed with an Amersham imager. NGS sequencing was performed on a NovaSeq 6000 (Illumina). mRNA levels were quantified in real time using the Quant Studio™ 7 Flex Real-Time PCR System (Applied Biosystems). Cytokine levels were measured using the MILLIPLEX Analyzer with the xPONENT software.
Data analysis	Validation to check gRNA presence and representation was performed using calc_auc_v1.1.py (https://github.com/mhegde/) and count_spacers.py. CRISPR KO screens were analyzed using Mageck-Vispr v0.5.7. The top gene hits along with their significance from the CRISPR screen were visualized using a volcano plot and an RRA score plot using MAGeCKFlute v2.0.0. For immunoblotting, images were analyzed with ImageJ (v1.53a). Graphpad Prism version 7.0 and IncuCyte (v2022RevB) were used for data analysis.

For manuscripts utilizing custom algorithms or software that are central to the research but not yet described in published literature, software must be made available to editors and reviewers. We strongly encourage code deposition in a community repository (e.g. GitHub). See the Nature Portfolio guidelines for submitting code & software for further information.

Data

Policy information about availability of data

All manuscripts must include a data availability statement. This statement should provide the following information, where applicable:

- Accession codes, unique identifiers, or web links for publicly available datasets
- A description of any restrictions on data availability
- For clinical datasets or third party data, please ensure that the statement adheres to our policy

Next-generation sequencing results from the CRISPR screen are deposited in BioProject: PRJNA1009133. All other datasets are included in the published article and the supplementary information. The uncropped western blots are included in supplementary information (Supplementary Figs. 4-6). The numerical data points for the graphs are provided in Supplementary Data 1. All other data are available from the corresponding authors upon reasonable request.

Research involving human participants, their data, or biological material

Policy information about studies with human participants or human data. See also policy information about sex, gender (identity/presentation), and sexual orientation and race, ethnicity and racism.

Reporting on sex and gender	N/A
Reporting on race, ethnicity, or other socially relevant groupings	N/A
Population characteristics	N/A
Recruitment	N/A
Ethics oversight	N/A

Note that full information on the approval of the study protocol must also be provided in the manuscript.

Field-specific reporting

Please select the one below that is the best fit for your research. If you are not sure, read the appropriate sections before making your selection.

☒ Life sciences ☐ Behavioural & social sciences ☐ Ecological, evolutionary & environmental sciences

For a reference copy of the document with all sections, see nature.com/documents/nr-reporting-summary-flat.pdf

Life sciences study design

All studies must disclose on these points even when the disclosure is negative.

Sample size	Key experiments were repeated by independent researchers. If all 3 replicates gave similar results, experiments were considered as reproducible and completed. Prior sample size determination was not done.
Data exclusions	ROUT test with Q-value of 0.1 as a cut-off was used to exclude outliers. The data exclusion criterion was pre-established. All data were retained if removing outliers could have resulted in a sample size of less than 3.
Replication	Each experiment was performed with at least 2-3 biological replicates. All the reported results are from experiments in which every repeat gave similar results.
Randomization	For in vitro experiments, cells from the same pool of iBMDMs were randomly split into separate wells and subjected to the treatments.
Blinding	Investigators were not blinded. None of the reported experiments require subjective decision making. Key experiments were repeated by independent researchers. Therefore, there was no need for blinding.

Reporting for specific materials, systems and methods

We require information from authors about some types of materials, experimental systems and methods used in many studies. Here, indicate whether each material, system or method listed is relevant to your study. If you are not sure if a list item applies to your research, read the appropriate section before selecting a response.

Materials & experimental systems

n/a	Involved in the study
☐	☒ Antibodies
☒	☐ Eukaryotic cell lines
☒	☐ Palaeontology and archaeology
☒	☐ Animals and other organisms
☒	☐ Clinical data
☒	☐ Dual use research of concern
☒	☐ Plants

Methods

n/a	Involved in the study
☒	☐ ChIP-seq
☒	☐ Flow cytometry
☒	☐ MRI-based neuroimaging

Antibodies

Antibodies used

Immunoblotting: anti-caspase-1 (AdipoGen, AG-20B-0044, 1:1000), anti-caspase-7 (CST, #9492, 1:1000), anti-cleaved caspase-7 (CST, #9491, 1:1000), anti-caspase-8 (CST, #4927, 1:1000), anti-cleaved caspase-8 (CST, #8592, 1:1000), anti-pMLKL (CST, #37333, 1:1000), anti-MLKL (Abcepta, AP142728, 1:1000), anti-GSDMD (Abcam, ab209845, 1:1000), anti-GSDME (Abcam, ab215191, 1:1000), anti-GAPDH (Santacruz, sc-166574 HRP, 1:10,000), NSP9 of MHV (Rockland Immunochemicals, 200-301-A56, 1:1000); secondary anti-rabbit (111-035-047), anti-mouse (315-035-047) HRP antibodies from Jackson ImmunoResearch Laboratories.

Validation

Antibodies were validated by their source company.
anti-caspase-1 (AdipoGen, AG-20B-0044): Measuring the inflammasome: O. Gross; Methods Mol. Biol. (2012). https://adipogen.com/ag-20b-0042-anti-caspase-1-p20-mouse-mab-casper-1.html
anti-caspase-7 (CST, #9492): RIPK1 Distinctly Regulates Yersinia-Induced Inflammatory Cell Death, PANoptosis: R K Subbarao Malireddi; Immunohorizons (2020). https://www.cellsignal.com/products/primary-antibodies/caspase-7-antibody/9492
anti-cleaved caspase-7 (CST, #9491): RIPK1 Distinctly Regulates Yersinia-Induced Inflammatory Cell Death, PANoptosis: R K Subbarao Malireddi; Immunohorizons (2020). https://www.cellsignal.com/products/primary-antibodies/cleaved-caspase-7-asp198-antibody/9491
anti-caspase-8 (CST, #4927): Caspase-6 Is a Key Regulator of Innate Immunity, Inflammasome Activation, and Host Defense: Min Zheng; Cell (2020). https://www.cellsignal.com/products/primary-antibodies/caspase-8-antibody-mouse-specific/4927
anti-cleaved caspase-8 (CST, #8592): Caspase-6 Is a Key Regulator of Innate Immunity, Inflammasome Activation, and Host Defense: Min Zheng; Cell (2020). https://www.cellsignal.com/products/primary-antibodies/caspase-8-antibody-mouse-specific/8592
anti-pMLKL (CST, #37333): Caspase-6 Is a Key Regulator of Innate Immunity, Inflammasome Activation, and Host Defense. Min Zheng; Cell (2020). https://www.cellsignal.com/products/primary-antibodies/phospho-mlkl-ser345-d6e3g-rabbit-mab/37333
anti-MLKL (Abcepta (formerly Abgent), AP14272B, 1:1000): ZBP1-dependent inflammatory cell death, PANoptosis, and cytokine storm disrupt IFN therapeutic efficacy during coronavirus infection. Rajendra Karki; Sci Immunol (2022). https://www.abcepta.com/products/AP14272b-Mouse-Mlkl-Antibody-C-term
anti-GSDMD (Abcam, ab209845): Caspase-6 Is a Key Regulator of Innate Immunity, Inflammasome Activation, and Host Defense: Min Zheng; Cell (2020). https://www.abcam.com/gsdmd-antibody-epr19828-ab209845.html
anti-GSDME (Abcam, ab215191): Structural Mechanism for GSDMD Targeting by Autoprocessed Caspases in Pyroptosis: Kun Wang; Cell (2020). https://www.abcam.com/dfna5gsdme-antibody-epr19859-n-terminal-ab215191.html
anti-GAPDH (Santacruz, sc-166574 HRP): TAZ links exercise to mitochondrial biogenesis via mitochondrial transcription factor A: Jun-Ha Hwang; https://www.scbt.com/p/gapdh-antibody-h-12
NSP9 of MHV (Rockland Immunochemicals, 200-301-A56): https://www.rockland.com/categories/primary-antibodies/hepatitis-virus-a59-nonstructural-protein-9-antibody-200-301-A56/
